# NIH research funding and early career physician scientists: continuing challenges in the 21st century

**DOI:** 10.1096/fj.13-241687

**Published:** 2014-03

**Authors:** Howard H. Garrison, Anne M. Deschamps

**Affiliations:** Office of Public Affairs, Federation of American Societies for Experimental Biology, Bethesda, Maryland, USA

**Keywords:** science policy, workforce, training

## Abstract

Physician scientists (researchers with either M.D. or M.D.-Ph.D. degrees) have the unique potential to combine clinical perspectives with scientific insight, and their participation in biomedical research has long been an important topic for policymakers and educators. Given the recent changes in the research environment, an update and extension of earlier studies of this population was needed. Our findings show that physician scientists are less likely to take a major role in biomedical research than they were in the past. The number of physician scientists receiving postdoctoral research training and career development awards is at an all-time low. Physician scientists today, on average, receive their first major research award (R01 equivalent) at a later age than in the 1980s. The number of first-time R01-equivalent awards to physicians is at the same level as it was 30 yr ago, but physicians now represent a smaller percentage of the grant recipients. The long-term decline in the number of physicians entering research careers was temporarily halted during the period of substantial U.S. National Institutes of Health (NIH) budget growth (1998–2003). These gains are lost, however, in the subsequent years when NIH budgets failed to keep pace with rising costs.— Garrison, H. H., Deschamps, A. M. NIH research funding and early career physician scientists: continuing challenges in the 21st century.

Physician scientists are uniquely capable of asking clinically relevant questions in research settings and bringing rigorous scientific inquiry to the care of patients. As such, they are a vital component of the biomedical research workforce (1). In the past decade, U.S. National Institutes of Health (NIH) grants to investigators with M.D. degrees were twice as likely to involve research with human subjects than were grants to scientists with Ph.D. degrees (2). Grants to researchers with both M.D. and Ph.D. degrees were slightly more likely to involve human subjects than those awarded to scientists with a Ph.D. only.

In medicine, the desire to combine scientific observation with a clinical perspective has a long tradition, with roots going back to classical antiquity (3). Success reaching the goal, however, has varied over time and place, and for several decades the number of physician scientists [researchers with M.D. or M.D. and Ph.D. (M.D.-Ph.D.) degrees] has been a concern for U.S. educators and policymakers.

In 1979, Wyngaarden (4) initiated a dialog on the role of medical training in biomedical research by pointing to a sharp decline in the number of research training fellowships for M.D.s. The decline in physician scientists' participation in biomedical research was attributed by Gill (5) to economic and intellectual changes that made research careers less attractive for young physicians. Moody (6) pointed to cost-containment polices limiting opportunities for research in clinical settings. The growing debt burden of medical school graduates, the increased length of the postdoctoral training required for a successful research career, and the instability inherent in a NIH-funded research career were also identified by Rosenberg (7) as reasons for the declining population of physician scientists.

Building on the analytic model used by Wyngaarden (4) and others, Zemlo *et al.* (8) examined data on medical students (career plans and debt levels), faculty appointments, and research participation (major professional activities, NIH grant applications, and NIH research awards). This study, using data through the mid-1990s, revealed that medical students' intentions to pursue a research career plummeted, while average debt levels of new medical school graduates steadily increased. In addition, the number of M.D.s supported on NIH training and fellowship grants declined, and, while the total number of applications for NIH research funding was expanding, the number of first-time grant applications submitted by M.D.s did not increase (8).

It has been more than a decade since a comprehensive examination of the participation of physician scientists in the biomedical workforce was conducted, and during that time there have been dramatic changes in the research environment. Funding for NIH increased substantially in the late 1990s, doubling from $13.675 billion in 1998 to $27.167 billion in 2003 (9). In response, institutions significantly expanded their research capacity and training levels (10). The number of physicians applying for NIH R01 grants also rose in the early years of the 21st century, though not as rapidly as for applicants holding Ph.D. degrees (11).

Growth in the NIH budget, however, came to an abrupt, unexpected end in 2004 and remained static for the next decade. When adjusted for inflation, the purchasing power of the NIH budget declined after 2003. In 2013, the appropriation for NIH was $29.151 billion, but after adjusting for risings costs, it was 23% below the 2003 level (12). The recession of 2008 also reduced research funding from other sources, as institutional endowments and private funding for biomedical research shrank. Research expenditures by pharmaceutical companies, which had grown steadily since the mid-1990s, leveled off after 2007 (13).

The size and composition of the research workforce continues to be a critical concern for policymakers. Recently NIH undertook a major examination of the research workforce (14) and a separate investigation of diversity in the research workforce (15). Neither study, however, looked at physician scientists. Given the major changes in the research environment, and with the encouragement of program administrators at the Doris Duke Charitable Foundation, who used the results from the Zemlo *et al.* (8) study to develop training programs, we updated the earlier analysis to assess changes in the U.S. physician scientist workforce. We focused on the critical early career stages, examining data on career plans, research training, major professional activity, faculty positions, and first NIH research grants (16).

## ANALYSIS

Survey data and institutional records from several sources were used in this study. Longitudinal, deidentified data were obtained from the American Medical Association (AMA), the Association of American Medical Colleges (AAMC), and NIH. Data describing major professional activities of physicians were obtained from Medical Marketing Service Inc. (Schaumburg, IL, USA), which manages AMA data; annual editions of the *Physician Characteristics and Distribution in the US* (AMA); or the *AAMC Data Book*. Information on student interest in research careers was obtained from the annual AAMC Matriculating Student Questionnaire (MSQ) and Graduation Questionnaire (GQ). AAMC Data Services provided the aggregate responses to the following question on the MSQ and GQ: “How extensively do you expect to be involved in research during your medical career?” Those specifying either an exclusive or a significant involvement in research were deemed interested in a research career. Nonresponses were excluded from analysis. MSQ asked this question from 1987 through 2006, while the GQ asked this question from 1982 through 1996 and 2000 through 2011.

In response to Freedom of Information Act (FOIA) requests, the NIH Office of Extramural Research provided data for degree of postdoctoral trainee in Ruth L. Kirschstein NRSA Institutional Research Training Grant T32 programs for fiscal years 1982–2009; average age of first-time R01-equivalent awardees for fiscal years 1980–2011 by degree; and first-time R01 applications and awards for fiscal years 1982–2011 by degree. Average age of first time R01-equivalent awardees includes the R29 award in earlier years and the DP2 in 2008 and later. F32, K08, and K23 data were collected using the NIH Research Portfolio Online Reporting Tools (RePORT) Report Catalogue (http://report.nih.gov). Due to the large number of cases with missing information on doctorate degree prior to 1985, the analysis of R01-equivalent grants begins with 1985. An evaluation of the study design undertaken by the Federation of American Societies for Experimental Biology's Protection of Human Subjects Committee determined that the methods for reporting summary data qualified for Institutional Review Board exemption.

### Major professional activity

**Figure 1.**
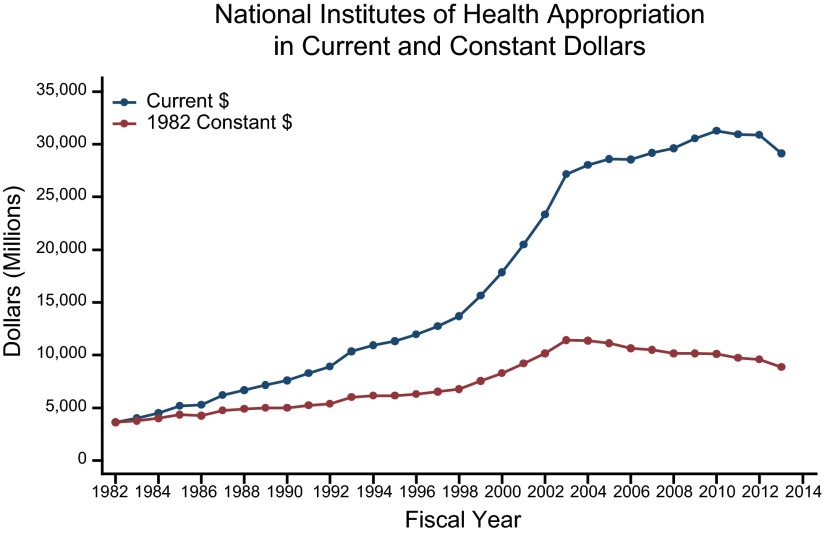
NIH appropriations in current and constant dollars from 1982 through 2013. Does not include American Recovery and Reinvestment Act funds.

**Figure 2.**
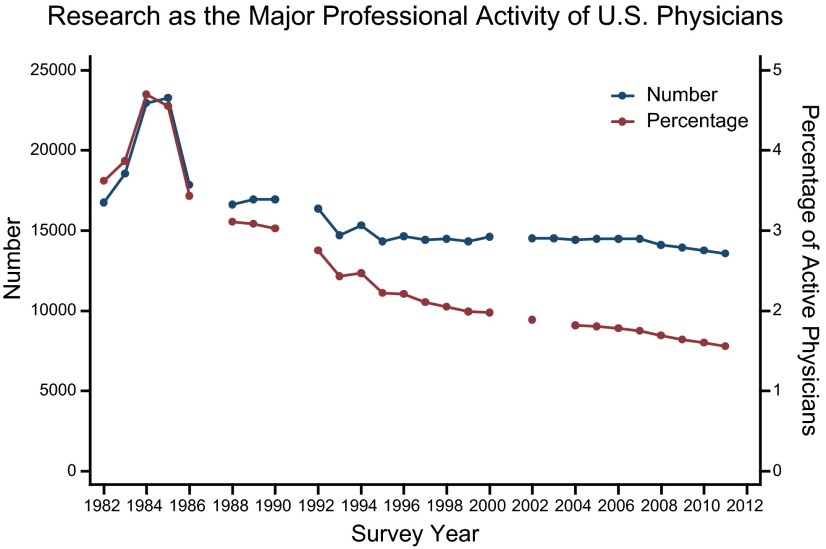
Number and percentage of U.S. physicians who report research as their major professional activity on the Physicians' Practice Arrangements questionnaire. The definition of research includes physicians in activities that develop new medical knowledge and includes physicians in research fellowships. Percentage is calculated using active physicians.

While NIH funding grew (**Fig. 1**) and the number of physicians in the United States has nearly doubled since 1980 (16), the number of U.S. physicians reporting research as their primary activity declined from 16,743 in 1982 to 13,557 in 2011. In 1982, 3.6% of U.S. physicians reported that their primary activity was research (**Table 1**). Since then, the percentage of U.S. physicians whose primary activity was research declined steadily. By 2011 only 1.6% of the physician population in the United States reported research as their major professional activity (**Fig. 2**).

**Table 1.** NIH research support, major professional activity of U.S. physicians, medical students' career intentions, and research training for physicians

Year	NIH funding ($10^6^)	U.S. physicians reporting research as major professional activity		Medical students with exclusive or significant interest in a research career (%)		Physicians supported on NIH T32 institutional training grants		NIH T32 institutional training grant positions held by physicians (%)		Physicians submitting NIH F32 postdoctoral research fellowship applications		NIH F32 postdoctoral research fellowship applications from physicians (%)		Physicians receiving NIH F32 postdoctoral research fellowship awards		NIH F32 postdoctoral research fellowship awards made to physicians (%)		K08 career development applications, clinical applicants		K23 career development applications, clinical applicants	
		*n*	%	M	G	MD	MD-PhD	MD	MD-PhD	MD	MD-PhD	MD	MD-PhD	MD	MD-PhD	MD	MD-PhD	S	A	S	A
1982	3,642	16,743	3.6		11.4	1421	220	35.1	5.4									110	46		
1983	4,024	18,535	3.9		13.1	1517	191	36.9	4.7									100	55		
1984	4,494	22,945	4.7		13.2	1518	212	37.6	5.3									200	94		
1985	5,149	23,268	4.6		14.5	1618	248	38.8	5.9									288	132		
1986	5,262	17,847	3.4		14.5	1673	267	40.2	6.4									420	119		
1987	6,183			13.8	14.7	1633	300	40.4	7.4									293	147		
1988	6,667	16,612	3.1	15.8	15.5	1634	303	40.9	7.6									239	126		
1989	7,145	16,941	3.1	15.0	15.8	1626	305	41.1	7.7									249	98		
1990	7,576	16,930	3.0	14.0	14.8	1635	311	38.8	7.4									294	149		
1991	8,277			11.3	13.7	1605	320	36.6	7.3									270	143		
1992	8,922	16,367	2.8	12.4	14.4	1624	311	36.9	7.1									304	157		
1993	10,336	14,716	2.4	12.2	13.7	1439	328	33.1	7.5									387	164		
1994	10,956	15,317	2.5	11.2	13.7	1399	397	31.5	8.9									452	213		
1995	11,300	14,340	2.2	11.0	12.3	1270	412	29.7	9.6									395	197		
1996	11,928	14,650	2.2	10.8	11.9	1258	398	29.2	9.2									506	298		
1997	12,741	14,434	2.1	9.4		1178	417	27.3	9.7									557	306		
1998	13,675	14,479	2.0	10.2		1227	355	27.8	8.1									536	311		
1999	15,629	14,333	2.0	9.7		1340	322	29.9	7.2									479	249		
2000	17,841	14,598	2.0	9.7	11.7	1469	321	32.5	7.1									496	250		
2001	20,459			10.3	11.9	1579	358	33.0	7.5									515	255		
2002	23,321	14,526	1.9	10.8	12.0	1676	362	33.6	7.3	171	37	11.0	2.4	73	20	11.9	3.3	560	293	421	196
2003	27,167	14,521		10.9	12.8	1699	369	32.5	7.0	197	61	10.1	3.1	69	20	9.7	2.8	592	280	505	214
2004	28,037	14,410	1.8	11.5	11.5	1742	347	32.9	6.6	207	67	9.3	3.0	56	31	7.8	4.3	669	267	635	226
2005	28,594	14,471	1.8	13.1	15.4	1709	317	33.1	6.1	250	56	10.5	2.3	72	13	10.1	1.8	676	266	679	232
2006	28,560	14,475	1.8	13.1	16.5	1735	279	33.9	5.5	212	76	8.3	3.0	48	29	6.8	4.1	635	215	666	180
2007	29,179	14,490	1.7		17.3	1728	273	33.6	5.3	204	65	8.4	2.7	56	15	8.9	2.4	524	189	650	217
2008	29,607	14,087	1.7		17.4	1694	266	33.5	5.3	162	50	7.5	2.3	55	15	8.6	2.3	509	222	574	216
2009	30,545	13,954	1.6		18.8	1640	251	32.9	5.0	153	44	7.6	2.2	44	14	8.2	2.6	466	221	517	227
2010	31,238	13,755	1.6		16.8			31.9	4.7	179	42	7.7	1.8	50	10	7.7	1.5	480	211	558	211
2011	30,916	13,557	1.6		17.1					129	34	5.6	1.5	35	14	5.8	2.3	425	177	599	203

M, matriculating; G, graduating; S, submitted; A, awarded.

### Student interest in research careers

**Figure 3.**
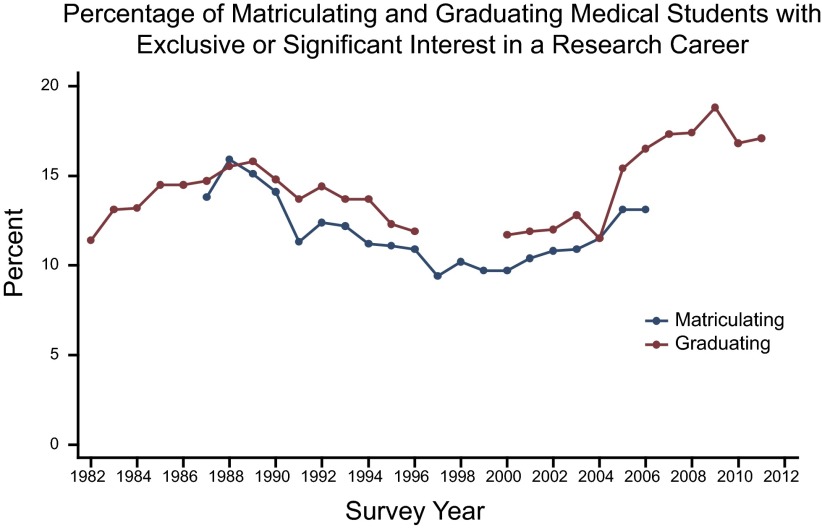
Information on student interest in a research career was obtained from the AAMC MSQ and GQ, which survey these student populations annually. Aggregate responses to the question on the MSQ and GQ “How extensively do you expect to be involved in research during your medical career?” were collected. Those specifying either an exclusive or a significant involvement in research were deemed interested in a research career. Nonresponses were excluded from analysis. MSQ asked this question from 1987 through 2006, while GQ asked this question from 1982 through 1996 and 2000 through 2011.

The percentages of matriculating and graduating medical students with strong (“exclusive” or “significant”) interest in a research career tended to move in tandem, rising during the period of rapid increases in NIH appropriations (**Fig. 3**). In 1988, nearly 16% of the matriculating medical students reported strong interest in a research career (Table 1). This fraction decreased in subsequent years, and, by 1997, only 9.4% indicated strong interest in a research career. In 1998, plans for research careers began to rise among matriculating medical students. By 2005, 13.1% had an interest in pursuing a research career.

A similar pattern was found among graduating medical students (Fig. 3). Aspiration for a career in research declined from 1988 through 1996. Individuals who were in medical school from 2000 through 2004, during the era of rising NIH budgets, however, had higher levels of aspiration for a research career. Research aspirations for graduates continued to rise and reached 18.8% in 2009, when the American Recovery and Reinvestment Act provided NIH with an additional $10 billion in research funding.

### Postdoctoral research training

Formal postdoctoral research training has become a necessary step in the path to an independent research career in the biomedical sciences, and there are several ways for physicians to receive this training. Historical data on the number of physicians receiving research training are available for two NIH-funded mechanisms: the T32 institutional training grants and the F32 individual fellowship awards. As was the case for medical student career plans, the number of physicians undertaking postdoctoral research training rose when NIH research funding increased substantially and tapered off when growth in NIH funding subsided.

**Figure 4.**
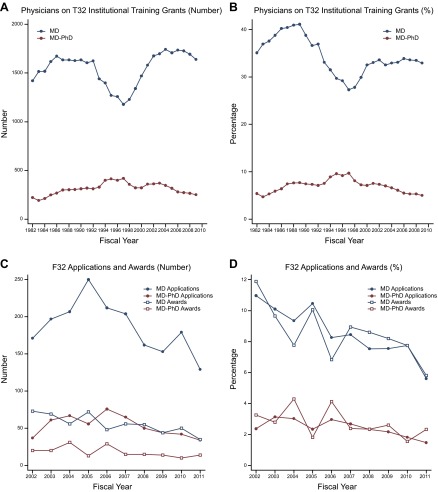
Number and percentage of physicians in postdoctoral fellowships. *A*) Data for the number of M.D.s and M.D.-Ph.D.s on postdoctoral NIH T32 Institutional Research Training grants from 1982 through 2009. T32 data include awardees receiving funding under the American Recovery and Reinvestment Act of 2009. Data were provided by NIH through FOIA requests. *B*) Data for percentage of physicians on T32 Institutional Research Training grants from 1982 to 2009. Percentages were calculated based on total number of trainees on T32 grants per fiscal year. *C*) Data for number of applications and awards for NIH F32 Individual Research Training grants from 2002 through 2011. Data were collected using the NIH RePORT Report Catalog. *D*) Data for percentage of physicians on F32 Individual Research Training grants from 2002 to 2011. Percentages were calculated based on total number of applications received and awards given per fiscal year.

The number of M.D.s in T32 training programs rose from 1421 in 1982 to 1624 in 1992, but then decreased each year from 1993 through 1997 (Table 1 and **Fig. 4*A***). Increased participation by M.D.-Ph.D.s offset some (but not all) of the loss of M.D.s. In the years during which NIH had substantial budget increases (1998–2003), the number of M.D.s on T32 grants rose once again. Starting in 2004, the number of T32 training grant slots held by both groups of physicians declined.

The percentage of T32 positions held by physicians reflected a similar pattern (Table 1 and Fig. 4*B*). Comprising nearly one-half of the T32 population in the late 1980s, the combined share of M.D. and M.D.-Ph.D. traineeships fell to just over one-third by 2010. Increased percentages of M.D.s on T32 training grants during the years of double-digit NIH budget growth reversed the decline for several years, but in recent years the percentage of T32 positions held by physicians began to decline again.

F32 fellowship awards to M.D.s have declined since the 1980s. Zemlo *et al.* (8) previously reported that F32 awards to M.D.s declined from 314 in 1985 to 180 in 1997, with most of the loss coming after 1993. During the era of large funding increases for NIH, the number of awards to M.D.s for individual postdoctoral fellowship awards grew in response to the rising number of F32 applications. Applications reached a peak in 2005 for M.D.s and in 2006 for M.D.-Ph.D.s before tapering off dramatically in subsequent years (Table 1 and Fig. 4*C*). By 2011, the number of F32 applications from individuals with medical degrees had fallen by nearly one-half. (Applications from Ph.D. scientists also increased during the early years of the 21st century but did not decrease as sharply in the postdoubling era as did those from M.D.s and M.D.-Ph.D.s; ref. 16.)

In recent years, physician scientists have also made up a diminished percentage of the F32 applicants and awardees (Table 1 and Fig. 4*D*). M.D.s made up 11.0% of the F32 applicants in 2002, but only 5.6% in 2011. M.D.-Ph.D.s declined from 2.4% to 1.5% from 2002 through 2011. The share of F32 awards to physician scientists also declined over this period, reflecting the trends in F32 applications.

### Career development awards

NIH offers a series of career development awards to assist early career scientists holding clinical doctorates initiate an independent program of research. K08 awards (Mentored Clinical Scientist Research Career Development Awards), in existence since the early 1970s, provide support and “protected time” (release time from other institutional responsibilities) for an intensive, supervised research career development experience to individuals with a clinical doctorate degree. Another mechanism, the K23 award (Mentored Patient-Oriented Research Career Development Awards), was established in 1999 to provide protected time for individuals with a clinical doctorate who are interested in pursuing careers in patient-oriented research.

Applications for K08 awards grew steadily after the inception of the program in the early 1970s and continued through 2003, the last year of substantial growth in the NIH budget. When growth in NIH research funding ended and the NIH budget lost ground in constant (inflation-adjusted) dollars, the number of K08 applications dropped by one-third.

**Figure 5.**
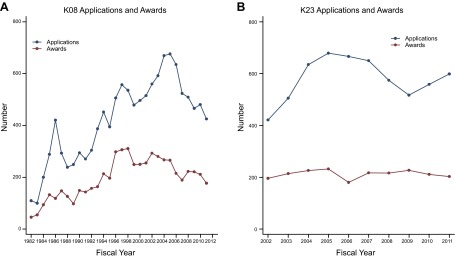
Early career grant application and awards for clinical doctorate degree holders. *A*) Data for the number of applications and awards for K08 Mentored Clinical Scientist Research Career Development Awards from 1982 through 2011. *B*) Data for the number of applications and awards for K23 Mentored Patient-Oriented Research Career Development Awards from 2002 through 2011. K08 and K23 data were collected using the NIH RePORT Report Catalog and from earlier transmissions from NIH.

In recent years, K08 awards have also fallen sharply. In 2002, NIH made 293 K08 awards. In 2011, there were only 177 (Table 1 and **Fig. 5*A***). For most of the program's history, the number of K08 awards reflected the number of applications, with approximately half of the applicants receiving awards. After 2003, however, the number of K08 awards declined at a faster rate than the number of applications, and applicant success rates fell below 40% from 2004 through 2007.

K23 applications rose from 421 in 2002 to 679 in 2005 before declining in each of the next 4 yr (Table 1 and Fig. 5*B*). There were 599 applicants in 2011, 80 fewer than in 2005. The number of K23 awards, however, remained constant throughout the program's existence at ∼200 awards/yr (Fig. 5*B*).

### NIH research grants

In most U.S. academic research settings, a large, multiyear grant is needed to establish a laboratory, initiate a sustained program of independent research, and make a substantial contribution to biomedical science. NIH R01 grants are the major source of such support. In addition to the R01 mechanism, NIH has used other mechanisms to provide multiyear support for discrete projects, including R29 First Independent Research Support and Transition (FIRST) awards for early career investigators and R37 Method to Extend Research in Time (MERIT) awards for highly productive scientists. These grants, along with the Director's Pioneer (DP1) awards, are collectively referred to as “R01-equivalent” grants. At many academic institutions, an individual must have one or more R01-equivalent grants to qualify for tenure and promotion. In 2011, the average R01 grant had a budget of $408,600 (including indirect costs; ref. 17).

R01 grants are awarded to experienced investigators to continue work on existing projects (“competitive renewals”) or to begin research on new subjects (type 1 awards). Approximately one-fourth of all new (*i.e*., type 1) R01 awards are “first-time” R01 grants, awards made to applicants who had not previously received R01-equivalent funding (18). Change in the distribution of first-time R01-equivalent awards is a highly sensitive, leading indicator of the future composition of the research workforce.

**Figure 6.**
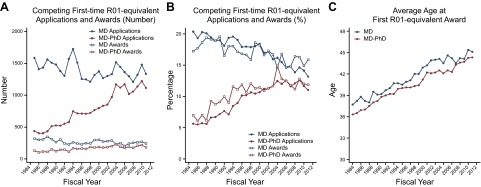
First major grant applications and awards for M.D.s and M.D.-Ph.D.s. *A*) Data for number of first-time R01-equivalent applications and awards for M.D.s and M.D.-Ph.D.s from 1985 through 2011. Due to the large number of cases with missing information on doctorate degree, the analysis of R01-equivalent grants does not use data between1982 and 1984 and begins with 1985. *B*) Data for percentage of applications and awards for M.D.s and M.D.-Ph.D.s from 1985 to 2011. Percentages were calculated based on the total number of applications and awards per fiscal year. *C*) Data for average age of first-time R01 equivalent awardees from 1982 through 2011. Average age of first-time R01-equivalent awardees includes the R29 award in earlier years and the DP2 in 2008 and later.

The number and percentage of first-time R01-equivalent awards to M.D.s reached its peak in the late 1980s (**Table 2** and Fig. 6). In 1988, M.D.s received 345 first-time R01 awards, 19.4% of the total. In 1996, they received only 228 (16.8% of the total). During the period of rapid budget growth for NIH, the situation improved modestly, and, in 2000, there were 299 first-time R01-equivalent awards to M.D.s. The percentage of grants going to M.D.s rebounded as well. But after 2004 the number of first-time R01-equivalent awards to M.D.s fell (Table 2 and **Fig. 6*A***). By 2011, M.D.s received only 243 first-time R01-equivalent awards, 15.9% of the total.

**Table 2.** R01-equivalent applications and awards for physician scientists

Year	First-time R01-equivalent applications submitted by physicians				First-time R01-equivalent awards to physicians				Average age at first R01-equivalent grant award	
	*n*		%		*n*		%			
	MD	MD-PhD	MD	MD-PhD	MD	MD-PhD	MD	MD-PhD	MD	MD-PhD
1985	1585	437	20.4	5.6	318	129	17.2	7.0	38.2	37.0
1986	1409	403	19.3	5.5	298	102	17.7	6.1	38.0	37.5
1987	1434	404	20.3	5.7	308	116	18.6	7.0	39.5	38.0
1988	1559	435	20.1	5.6	345	110	19.4	6.2	39.1	38.2
1989	1507	513	19.4	6.6	310	150	19.1	9.3	39.2	38.8
1990	1482	525	18.9	6.7	263	122	18.9	8.8	39.7	39.0
1991	1379	520	19.0	7.1	305	142	19.6	9.1	40.0	39.2
1992	1302	551	18.1	7.7	243	147	16.5	10.0	40.7	39.2
1993	1570	578	19.3	7.1	229	114	18.0	9.0	40.7	39.9
1994	1725	746	19.5	8.5	262	168	18.0	11.6	40.5	40.0
1995	1512	712	19.0	8.9	243	158	17.1	11.1	40.9	40.1
1996	1253	710	17.9	10.1	228	161	16.8	11.9	41.1	40.1
1997	1249	709	18.0	10.2	247	158	16.6	10.6	42.0	40.3
1998	1212	722	17.9	10.6	245	186	15.8	12.0	42.0	40.4
1999	1317	764	18.0	10.5	297	180	18.6	11.3	42.9	41.2
2000	1374	797	18.4	10.7	299	188	18.2	11.4	43.2	42.2
2001	1266	835	17.0	11.2	271	178	16.6	10.9	43.9	42.1
2002	1300	853	17.1	11.2	279	183	17.3	11.3	44.0	42.2
2003	1330	971	15.9	11.6	288	201	16.7	11.7	44.1	42.5
2004	1515	1167	16.2	12.5	230	235	14.6	14.9	43.5	42.1
2005	1442	1120	15.5	12.0	248	189	16.8	12.8	44.6	42.5
2006	1362	1171	14.6	12.5	204	173	14.7	12.5	44.2	42.3
2007	1298	1020	14.7	11.6	242	193	14.8	11.8	43.5	43.3
2008	1210	1056	13.9	12.1	253	172	16.4	11.1	44.2	43.6
2009	1318	1136	14.6	12.6	261	205	16.3	12.8	44.1	43.7
2010	1478	1219	14.1	11.6	270	218	14.9	12.0	45.4	44.3
2011	1332	1114	13.1	11.0	243	182	15.9	11.9	45.1	44.3

First-time R01 awards to M.D.-Ph.D.s grew steadily from 129 in 1985 to a high of 235 in 2004 (Table 2 and Fig. 6*A*). Combining data for M.D.s and M.D.-Ph.D.s, there were 425 first-time R01-equivalent awards to medically trained investigators in 2011 (27.8% of the total), a level comparable to that for the years immediately before and after the period of rapid NIH budget growth.

The decline in the number of first-time R01-equivalent grants to M.D.s was a result of a decline in applications. The percentage of M.D. applications funded (*i.e.*, the success rate) was very stable from 1985 through 2011, averaging just over 19%. In the late 1980s M.D.s submitted over 1500 R01-equivalent applications per year (∼20% of the total). In 2011 M.D.s submitted 1332 first-time R01-equivalent applications, 13.1% of the total.

Again, as was the case for T32 training position, a rising number of first-time R01-equivalent applications from M.D.-Ph.D.s partially offset the loss from M.D.s. Applications from M.D.-Ph.D.s rose substantially from 437 in 1985 to 1114 in 2011. M.D.-Ph.D.s comprised 5.6% of the applicant pool in 1985 and 11.0% in 2011. Applications from physician scientists with either M.D. or M.D.-Ph.D. degrees accounted for 26.0% of the total in 1985 and 24.1% in 2011.

Increased competition for R01 grants has also affected the timing of first awards. In 1985, the average age at which an M.D. was awarded his or her first R01-equivant grant was 38.2 yr (Table 2). This was more than 1 yr older than the average age for first-time R01-equivalent grantee with M.D.-Ph.D. degrees (37.0 yr). By 2011, the average age at first R01 was 7 yr higher for both groups, reaching 45.1 yr for M.D.s and 44.3 yr for M.D.-Ph.D.s. During the 5 yr of double-digit growth for NIH, however, the rise in age at first R01-equivalent grant slowed somewhat for all physician scientists (Fig. 6*C*).

## DISCUSSION

Combining both medical and scientific expertise, physician scientists have tremendous potential to advance biomedical research and improve the translation of new discoveries into health care advances. Their level of involvement in biomedical research is, therefore, a topic of great interest to educators, research institutions, and policymakers. Building on earlier analyses (4, 8, 11), we found that in 2011 physicians were less likely to be involved in biomedical research than they were in the past.

The study used records from AMA, AAMC, and NIH to analyze the change over time in the research activities of early career physicians. Each measure captures a specific aspect of the research career, and some indicators have limitations. Survey nonresponse may bias the findings from the AMA Record of Physicians Professional Activity questionnaire. The records for NIH R01-equivalent grants are incomplete measures of research involvement; there are additional sources of research funding including other government agencies, private foundations, institutional resources, patient-donors, and private industry. Moreover, physician scientists make valuable, substantive contributions to research in ways other than as a principal investigator. Many collaborate on grants awarded to other researchers, working as part of a larger team. They may also serve as consultants and advisors, making substantive contributions to a wide range of projects. We cannot assess the total volume of research contribution made by physician scientists, but we have clear evidence that the nature of their involvement in research has changed substantially; today early career physicians make up a smaller share of the independent investigators in biomedical research than they had in the past.

A declining number of physicians report research as their major professional activity. This pattern is likely to continue, as the training pipeline for future physician scientists is also shrinking. Fewer physicians are applying for (and receiving) postdoctoral research fellowships, and the number of training grant slots filled by M.D.s is decreasing. Some of the long-term decline in career plans of medical students and career development grant applications was reversed by the increase in NIH funding during the late 1990s and early years of the 21st century. When growth in NIH research funding ended, and funding decreased in terms of purchasing power, research career plans and training activities declined again.

Debt levels for U.S. medical school graduates grew steadily from the early 1980s through 2008 (19). The rising cost of medical education and the growing indebtedness of new graduates may be discouraging physicians from pursuit of research careers. An extended period of research training will suppress earnings for several years, and the lower salaries characteristic of academic medicine make loan repayment much harder. Moreover, the terms of academic employment, with the increased dependence on grant funding for salaries and job security, add an element of uncertainty to an equation that is already weighted against a research career (20). In 2000, NIH substantially expanded its loan repayment program for certain categories of researchers, but there is no evidence that this effort had made a measurable impact on the supply of physician scientists. Andriole *et al.* (21) found that M.D.s with higher levels of debt at graduation were less likely to have faculty positions.

The relationship between debt and faculty position, however, is a complex one. Debt was not significant in multivariate models that included other variables, suggesting that the effect of debt is a complex one that is correlated with other career choices and demographic characteristics (21). In a study limited to M.D.-Ph.D. graduates, however, Jeffe and Andriole (22) found that the relationship between indebtedness and faculty positions remained significant after other variables were considered. Fang (23), on the other hand, found that there was a relationship between debt and faculty appointments only for 1992 and 1993 medical school graduates, and he found no relationship for earlier graduates (with lower average debt levels).

The declining number of physicians applying for K awards may be related to financial constraints affecting medical schools and research hospitals. K awards require that the grantee receive 75% protected time for research. Due to legislative, budgetary, and programmatic constraints, K awards are often unable to pay 75% of the physician's salary, requiring substantial cost sharing by the institution or the investigator.

Budgetary constraints in the form of rising costs and declining program budgets have affected the number of K awards. Prior to 1998, the number of K08 awards grew in direct proportion to the number of K08 applications. This is no longer the case. Budget increases for the K08 program ended in 2003, and the total program budget has declined since that date. At the same time, the average cost of a K08 award (which includes funding for salaries as well as for research expenses) has increased, rising from $95,000 in 1998 to $134,000 in 2005 (16). As a result, the number of awards declined sharply. In 2011, only 177 K08 awards were made, 116 fewer than in 2002.

In recent years, applications for R01-equivalent grants have increased faster than the growth in NIH research grant funding. Intensified competition for NIH R01-equivalent awards has not only reduced the likelihood of applicant success, but has also extended the amount of time that early career scientists spend seeking funding for an independent laboratory (24). The extended period of preparation required for an independent career in biomedical research, coming after a lengthy period of medical education, must be seen as an impediment to the pursuit of this vocation.

A prolonged research apprenticeship is costly to individuals, educational institutions, research sponsors, and the public. For prospective scientists, the additional preparation time and expense creates a disincentive to pursue a career in research. For society as a whole, there is the loss of potential scientists and the knowledge that they generate. Higher training costs mean less funding available for research projects, and the delayed onset of a research career shortens the expected work life of a researcher. Taken together, these factors slow the advancement of biomedical science.

Although we focus on early career activities, problems are not limited to the entry level. The dedicated and talented individuals who survive these obstacles and successfully initiate a career in biomedical research still face major challenges at later stages, and attrition among the ranks of physician scientists is a concern. Dickler *et al.* (11), for example, reported that successful M.D. R01 applicants are less likely than Ph.D.s to apply for a second R01 grant. Even for the exceptional individuals who overcome the odds against initial success, the continuing challenges may result in shortened research careers.

In some cases, the loss of M.D. scientists is partially offset by increases for M.D.-Ph.D.s (*e.g.*, first-time R01-equivalent grant applications and awards). Although this reduces the decline of physician participation in research, the shift toward M.D.-Ph.D.s may affect the types of research being undertaken. NIH-funded researchers with M.D.-Ph.D.s are less likely than M.D.s to be engaged in human subjects research (2). Overreliance on M.D.-Ph.D.s for the physician scientist workforce will also exclude those who discover a passion for research after they have begun their undergraduate medical education.

Perhaps the most ominous finding is the relationship of research participation to economic factors and the implications for the future supply of physician scientists. Training decisions, training duration, and applications for research grants rise with increases in appropriations for NIH and decline when budget growth slows and purchasing power diminishes. While Teitelbaum (25) has expressed concern that training practices and early career decisions for Ph.D.s are not responsive to negative feedback from the job markets, this is not the case for physicians. Early career research involvement for M.D.s is highly sensitive to changes in NIH research funding.

Research funding, however, is not the only financial factor in the equation. Student debt is increasing as public funding for higher education wanes (26). Financial pressures on the research enterprise are widespread and growing. Medical schools and teaching hospitals are facing increased pressure to reduce operating costs. Proposed reductions in Medicare, Medicaid, and other reimbursement programs, such as the sequestration of funds mandated by the Budget Control Act of 2011, are a growing threat to the entire health care system. Discretionary budgets that enabled institutions to provide protected time for researchers have largely become a thing of the past as institutions become increasingly reluctant to support any activity that does not show near-term financial benefit. Without major change or intervention, these factors suggest that the prospects for relief from the trends in declining research involvement of early career physicians are extremely remote. The downward spiral observed in the analysis above is likely to be just the beginning. In 2013 sequestration took $1.5 billion from the NIH budget, and the government shutdown brought the research enterprise to a grinding halt. The future looks grim indeed.

How many physician scientists do we need? Like many questions about research investment, there is no single correct answer. But with the opportunities for progress before us and the vast amount of human suffering, “fewer” is certainly the wrong answer.
